# High Speed Ventral Plane Videography as a Convenient Tool to Quantify Motor Deficits during Pre-Clinical Experimental Autoimmune Encephalomyelitis

**DOI:** 10.3390/cells8111439

**Published:** 2019-11-14

**Authors:** Jiangshan Zhan, Vladislav Yakimov, Sebastian Rühling, Felix Fischbach, Elena Nikolova, Sarah Joost, Hannes Kaddatz, Theresa Greiner, Julia Frenz, Carsten Holzmann, Markus Kipp

**Affiliations:** 1 Institute of Anatomy, Rostock University Medical Center, 18057 Rostock, Germany; jiangshan.zhan@campus.lmu.de (J.Z.); vladislavvd15@gmail.com (V.Y.); sebastian.ruehling@tum.de (S.R.); felix@famfischbach.de (F.F.); evladimirovanikolova@gmail.com (E.N.); sarah.joost@med.uni-rostock.de (S.J.); hannes.kaddatz@uni-rostock.de (H.K.); theresa.greiner@uni-rostock.de (T.G.); julia.frenz97@gmx.de (J.F.); carsten.holzmann@med.uni-rostock.de (C.H.); 2 Institute of Anatomy II, Faculty of Medicine, LMU Munich, 80336 Munich, Germany

**Keywords:** DigiGait™, experimental autoimmune encephalomyelitis, multiple sclerosis, gait analysis

## Abstract

Experimental autoimmune encephalomyelitis (EAE) is the most commonly used multiple sclerosis animal model. EAE mice typically develop motor deficits in a caudal-to-rostral pattern when inflammatory lesions have already developed. However, to monitor more subtle behavioral deficits during lesion development (i.e., pre-clinical phase), more sophisticated methods are needed. Here, we investigated whether high speed ventral plane videography can be applied to monitor early motor deficits during ‘pre-clinical’ EAE. For this purpose, EAE was induced in C57BL/6 mice and gait abnormalities were quantified using the DigiGait™ apparatus. Gait deficits were related to histopathological changes. 10 out of 10 control (100%), and 14 out of 18 (77.8%) pre-clinical EAE mice could be evaluated using DigiGait™. EAE severity was not influenced by DigiGait™-related mice handlings. Most gait parameters recorded from day 6 post-immunization until the end of the experiment were found to be stable in control mice. During the pre-clinical phase, when conventional EAE scorings failed to detect any functional impairment, EAE mice showed an increased *Swing Time*, increased *%Swing Stride*, decreased *%Stance Stride*, decreased *Stance/Swing,* and an increased *Absolute Paw Angle*. In summary, DigiGait™ is more sensitive than conventional scoring approaches to study motor deficits during the EAE pre-clinical phase.

## 1. Introduction

Multiple sclerosis (MS) is an autoimmune, inflammatory, demyelinating disease of the central nervous system (CNS). On the histopathological level, MS lesions are characterized by large inflammatory plaques of white matter demyelination. Such focal inflammatory lesions are associated with oligodendrocyte destruction, reactive gliosis and axonal degeneration. The composition of established inflammatory infiltrates varies between patients and/or lesion stages but commonly includes CD8^+^ T-lymphocytes and macrophages. In addition to focal white matter lesions, gray matter demyelination and/or atrophy and diffuse white matter injury are frequently observed [1,2,3]. While the characteristics of established lesions are well investigated, how such lesions develop is less well understood. On one hand, it is discussed that early during the development of inflammatory MS lesions, autoreactive T- and B-cells invade the brain parenchyma, get reactivated, and promote the development of inflammatory demyelination. Other authors suggest, however, that the recruitment of peripheral immune cells is a secondary phenomenon, triggered by a local, brain intrinsic degenerative event [4,5]. For example, one post-mortem study has shown that early MS lesions are characterized by oligodendrocyte degeneration and microglia activation in the absence of overt peripheral immune cells [6]. Whatever is true, imaging studies clearly demonstrate that subtle CNS pathologies can be observed before symptoms become evident [7,8,9].

For the development of new therapeutic options in MS, several models are available and can be roughly broken down into the categories of autoimmune and non-autoimmune animal models. Experimental autoimmune encephalomyelitis (EAE) is the most commonly used animal model to study autoimmune-mediated aspects of the disease. In this model, experimental animals (commonly rodents) are immunized with a CNS-related antigen administered in a strong adjuvant, usually complete Freund′s adjuvant (CFA). Following immunization, antigens are phagocytized by local professional antigen-presenting cells, transported to local lymph nodes or the spleen, where they trigger the development of encephalitogenic T_h1_- and T_h17_-cell immune responses. This finally leads to inflammation within different CNS regions, mainly the spinal cord and the cerebellum [10]. On the behavioral level, this model is characterized by an ascending paralysis that begins in the tail and spreads to involve the hind limbs and, finally, fore limbs. Although different grading systems exist, the disease is usually rated on a scale ranging from grades 0–5. Grade 1 is assigned to mice that have lost tail tonicity, whereas grade 2 is assigned to mice that additionally show hind limb weakness. As the disease progresses, through grade 3 and 4, fore limb motor dysfunction additionally develops.

In MS, especially the early stages of lesion pathophysiology are poorly understood. Several studies were able to demonstrate that discrete histopathological changes occur within the brain parenchyma before acute inflammatory lesions become visible. Such changes include fibrinogen deposition [11], oligodendrocyte injury [6], focal microglia activation [12], and the downregulation of neuronal and oligodendrocyte marker gene expression [13]. In EAE, clinical symptoms are generally applied to mark the onset of disease, because this coincides with autoimmune effector CD4^+^ T-cell infiltration into the CNS parenchyma [14,15]. However, recent reports show that structural and functional changes take place within CNS tissues before the development of clinically overt symptoms. Such observed changes include, among others, the activation of endothelial cells and astrocytes [16], reductions in myelin gene expression [13], or altered glutamate transmission [17]. Furthermore, in vivo imaging studies nicely demonstrate intraluminal crawling of encephalitogenic T-cells [14] and perivascular clustering of microglia [18] prior to the onset of clinical symptoms. In line with the observation of changes in brain homeostasis prior to the development of overt, inflammatory lesions our group recently demonstrated that toxic damage to the oligodendrocyte-myelin unit not just leads to glia activation but at the same time triggers the recruitment of peripheral immune cells into the CNS in the predisposed host [4,5,19]. Together, these data strongly implicate that subclinical alterations take place in the CNS tissue during the development of EAE that might predispose it to immunopathology.

In recent years, an extensive body of literature has demonstrated benefits of early treatment of MS with disease modifying drugs. Specifically, research has shown that early treatment in relation to disease onset is associated with significantly improved physical and mental outcomes, including lower relapse rates and lower expanded disability status scale (EDSS) scores, both in the short- and long-term [20,21,22,23]. A better understanding of the pre-clinical pathological processes would allow the development of early and probably effective therapeutic options. This requires mechanistic studies during the largely invisible pre-clinical disease stage. While novel and sensitive imaging modalities are currently available to visualize pathological changes during pre-symptomatic EAE, appropriate modalities to measure functional deficits are still missing.

In this work, we aimed to investigate whether high speed ventral plane videography is appropriate for the quantification of pre-clinical functional deficits in EAE.

## 2. Materials and Methods

### 2.1. Animals

For this study, 10-week-old C57BL/6 female mice (n = 40) were purchased from Janvier Labs, Le Genest-Saint-Isle, France. All experimental procedures were approved by the Review Board for the Care of Animal Subjects of the district government (Regierung Oberbayern; reference number 55.2-154-2532-73-15; Germany). The mice were maintained in a pathogen-free environment with a maximum of five animals per cage and with *ad libitum* food and water. Cages were changed once per week and microbiological monitoring was performed according to the Federation of European Laboratory Animal Science Associations recommendations. Mice were acclimated at the housing conditions for at least one week before EAE induction.

### 2.2. EAE Induction, Disease Scoring, and Experimental Groups

To induce the formation of encephalitogenic T-cells in peripheral lymphatic tissues, the mice were subcutaneously immunized with an emulsion of myelin oligodendrocyte glycoprotein (MOG_35–55_) peptide dissolved in complete Freund’s adjuvant followed by intraperitoneal injections of pertussis toxin (PTx) in PBS on the day of and the day after immunization (Hooke Laboratories, Inc., Lawrence, USA) as previously published [4]. Disease severity was scored as follows: 1, The entire tail drops over the observer’s finger when the mouse is picked up by the base of the tail; 2, the legs are not spread apart but held close together when the mouse is picked up by the base of the tail, and mice exhibit a clearly apparent wobbly gait; 3, the tail is limp and mice show complete paralysis of hind legs (a score of 3.5 is given if the mouse is unable to raise itself when placed on its side); 4, the tail is limp and mice show complete hind leg paralysis and partial front leg paresis, and the mouse is minimally moving around the cage but appears alert and feeding; 5, the mouse is euthanized due to severe paralysis. The parameter “*disease onset*” was defined as the day post immunization when the first clinical deficit (see above) was observed. The parameter “*maximum disease score*” was defined as the highest clinical score, reached by a mouse at any time-point during the experiment. The parameter “*cumulative disease score*” was calculated by adding all clinical scores, registered during the experiment for a single mouse.

The following treatment groups were included: Control^DigiGait^ mice: Non-immunized mice were subjected to gait analyses; EAE^Only^ mice: EAE was induced by MOG_35-55_ immunization + CFA/PTx, but mice were not subjected to gait analyses; EAE^DigiGait^ mice: EAE was induced by MOG_35-55_ immunization + CFA/PTx, and mice were subjected to gait analyses starting at day 6 post immunization; PTx^DigiGait^ mice: Mice were injected with CFA and PTx, and mice were subjected to gait analyses starting at day 6 post immunization.

### 2.3. High Speed Ventral Plane Videography and Evaluation

**Figure 1:**
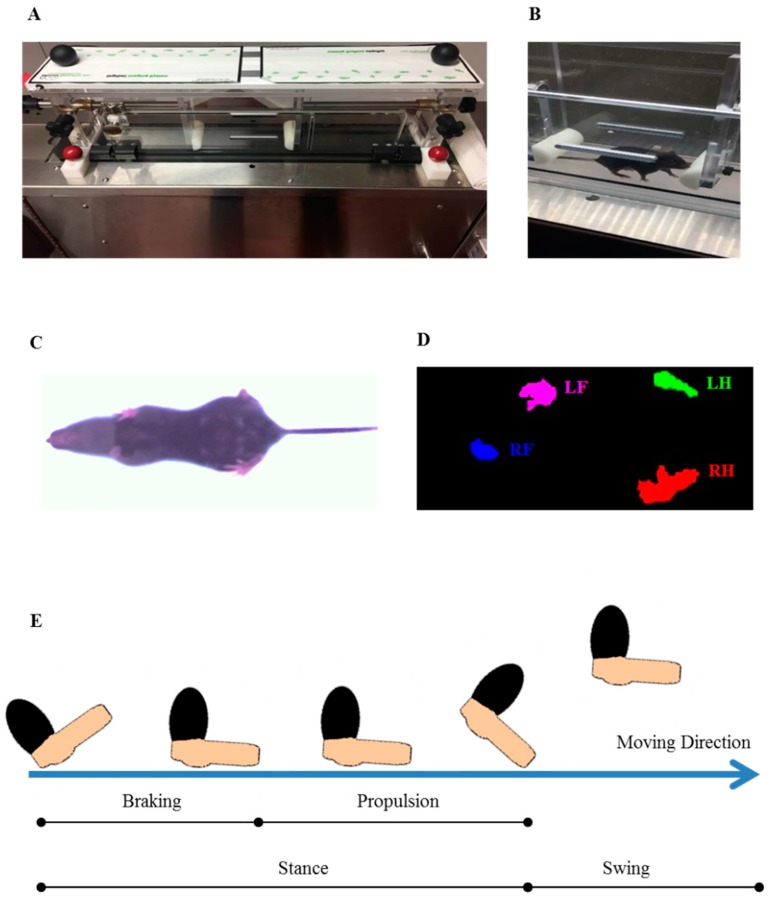
High speed ventral plane videography using the DigiGait™ setup. (**A**) Setup of the DigiGait™ imaging system. (**B**) DigiGait™ setup with a mouse in the running chamber during ventral plane videography recordings. (**C**) Representative image of a mouse during ventral plane videography recordings. (**D**) Representative image of the position of the single paws extracted from the ventral plane videography recordings by the provided analysis tool. (**E**) A graphical depiction of various aspects of a single mouse stride. Each stride can be subdivided into a stance and swing part. The stance part can be further subdivided into a braking and propulsion phase. LF: Left Fore; LH: Left Hind; RF: Right Fore; RH: Right Hind.

Gait parameters were assessed using the DigiGait™ imaging system along with the DigiGait™ 15.0 analysis software (Mouse Specifics, Inc.; Quincy, MA, USA) [24]. The DigiGait™ apparatus consists of a clear plastic treadmill with a high speed under-mounted digital camera (Basler Technologies Inc.) used for imaging paw prints. The treadmill belt was accelerated gradually to 15 cm/s. Images were collected at a rate of 140 frames/s. and stored as audio video interleaved (AVI) files for later blinded analyses. To improve the contrast for automated foot print analysis, the tails of the mice were colored with black dye. The treadmill belt was cleaned with 70% (*v/v*) ethanol between each animal testing. Animals were habituated to the machine one day prior to testing. Data obtained from the training day were not included in the final data evaluation. The image analysis software digitally encoded the individual paw area and position relative to the tread-belt. Each paw of the animal was treated as a unique signature so that later analyses of foot movements could be performed on separate limbs. Following this strategy, the DigiGait™ analysis software computes 39 gait parameters for the fore limbs and 43 for the hind limbs of each animal. The minimal duration of each video sequence required for subsequent foot-print analyses was 5 s. Runs where mice could not run at 15 cm/s for a minimum of 5 s were excluded from subsequent analyses. This number of strides has been validated as being sufficient to analyze treadmill walking behavior in mice [25]. Figure 1 illustrates the principal setup of the performed gait analyses.

To analyze gait abnormalities during the pre-clinical disease stage, we first quantified fore limb and hind limb gait patterns in five control and 10 EAE-induced mice. This first group is referred to as Cohort#1. To verify results of this first experiment, hind limb gait patterns were analyzed in another cohort of five control and 10 EAE-induced mice, referred to as Cohort#2. Both cohorts were finally evaluated by a second evaluator blinded to the treatment groups (i.e., Evaluator 2).

### 2.4. Rotarod Analysis

To determine balance and motor coordination in control (Control^Rotarod^; n = 10) and EAE (EAE^Rotarod^; n = 8) mice, an accelerod system (TSE Systems, Bad Homburg, Germany) for small rodents was used (TSE Systems, Bad Homburg, Germany) as previously published by our group [26]. The apparatus consists of a base platform and a rotating rod (30 mm diameter, 114 mm width) with a non-skid surface. Each experimental mouse was subjected to three training sessions at a constant rotation speed of 5 rpm (rounds per min) for 2 min. These training sessions were conducted from day 3 to day 5 post immunization. Data obtained from the training sessions were not included in the data evaluation. During the testing session, an accelerating modus was used, which began at 4 rpm and accelerated to 40 rpm over a period of 300 s (i.e., 5 min). Two trials per test day were carried out, with a 60 min rest in between each trial. For each trial and each animal, latency, maximum speed, and walking distance before falling off were automatically recorded. The testing sessions were repeated from day 6 to day 13 post immunization. Only data obtained during pre-clinical disease stages were included for the final data evaluation.

### 2.5. Tissue Preparation and Histological Evaluation

For (immuno-) histological studies, mice were deeply anaesthetized with ketamine (100 mg·kg^−1^ i.p.) and xylazine (10 mg·kg^−1^ i.p.), and transcardially perfused with ice-cold phosphate-buffered saline (PBS) followed by a 3.7% formaldehyde solution (pH = 7.4). Tissues were postfixed overnight in a 3.7% formaldehyde solution, dissected, and embedded in paraffin. 5 μm thick sections were prepared using a slide microtome, dried at ambient temperature for at least 3 h, and subsequently dried overnight at 48 °C before starting the different staining procedures. For immunohistochemistry, sections were rehydrated and, if necessary, antigens were unmasked by heating in a Tris/EDTA (pH 9.0) buffer. After washing in PBS, sections were blocked in blocking solution (serum of the species in which the secondary antibody was produced) for 1 h. Then, sections were incubated overnight (4 °C) with primary antibodies diluted in blocking solution. The next day, slides were incubated in 0.3% hydrogen peroxide/PBS for 1 h and then incubated with biotinylated secondary antibodies for 1 h followed by peroxidase-coupled avidin-biotin complex (ABC kit; Vector Laboratories, Peterborough, UK). Sections were finally exposed to 3,3′-diaminobenzidine (DAKO, Santa Clara, CA, USA) as a peroxidase substrate. To visualize cell nuclei, sections were briefly stained with hematoxylin solution if appropriate. Negative control sections without primary antibodies were processed in parallel to ensure specificity of the staining. For microglia labelling anti-ionized calcium-binding adapter molecule 1 antibodies ([IBA1] 1:5000; Wako; #019-19741) were combined with anti-rabbit secondary antibodies (1:200; Vector; #BA-1000). For lymphocyte labelling, anti-CD3 antibodies ([CD3] 1:500; Abcam; ab11089) were combined with anti-rat secondary antibodies (1:200; Vector; #BA 9400). Luxol fast blue (LFB)/periodic acid-Schiff (PAS) stains were performed following standard protocols. Stained and processed sections were digitalized using a Leica DM6 B automated microscope (Leica Microsystems CMS GmbH, Wetzlar, Germany) equipped with a DMC6200 camera.

**Figure 2:**
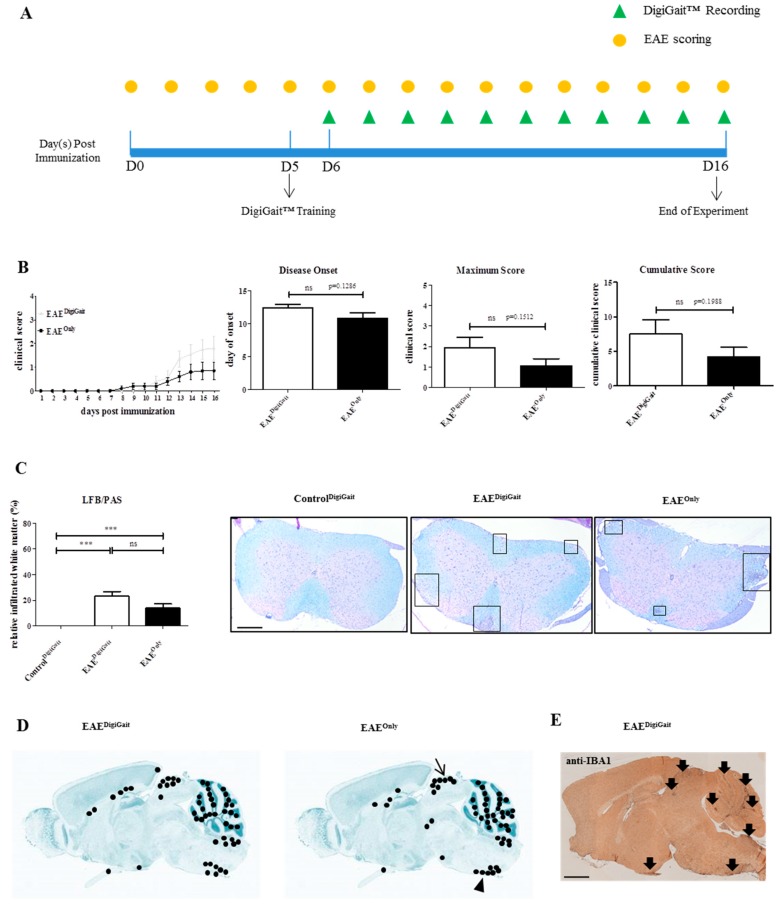
Manipulation during DigiGait™ recordings does not ameliorate EAE severity. (**A**) Schematic depiction of the experimental setup. D = days post immunization. The yellow circles indicate time points when EAE scoring was performed. The green triangles indicate time points when DigiGait^TM^-measurements were performed. Note that at day 5 post immunization (D5), one DigiGait^TM^ training session was conducted. (**B**) Clinical course and evaluation of the disease parameters *disease onset*, *maximum score,* and *cumulative score* in EAE^DigiGait^ (n = 10) and EAE^Only^ (n = 10) mice. Note that 8 EAE^DigiGait^ and 6 EAE^Only^ mice, which developed clinical disease, were included to calculate the parameter *disease onset*. Data from all mice were included to calculate the parameters *maximum score* and *cumulative score*. Statistical comparison was done using an unpaired t-test. (**C**) Extent of inflammatory demyelination among Control^DigiGait^, EAE^DigiGait^, and EAE^Only^ mice evaluated in LFB/PAS stained sections (n = 72 sections). Black boxes highlight the inflammatory foci. Statistical comparison was done using a one-way analysis of variance with the obtained *p*-values corrected for multiple testing using the Dunnett’s post hoc test. (**D**) Cumulative map of the spatial distribution of microgliosis in the CNS of EAE^DigiGait^ and EAE^Only^ mice, visualized by anti-IBA1 stains. Twenty sections from 10 individual animals were included per group. Each black dot shows the position of a focal IBA1^+^ lesion which was identified by both evaluators (J.Z. and H.K.). (**E**) Representative anti-IBA1 stain demonstrating IBA1^+^ lesions in an EAE^DigiGait^ mouse. Scale bar (**C**) = 300 µm; Scale bar (**E**) = 1 mm. EAE: Experimental Autoimmune Encephalomyelitis; LFB/PAS: Luxol fast blue/periodic acid-Schiff; CNS: Central Nervous System; IBA1: ionized calcium-binding adapter molecule 1. *** *p* ≤ 0.001, ns = not significant.

To analyse the extent of inflammatory demyelination in the spinal cord among Control^DigiGait^, EAE^Only^, and EAE^DigiGait^ mice, the entire white matter was outlined in the digitalized images of LFB/PAS stained sections, and the areas of infiltrated white matter were measured using the open source program ImageJ 1.50. The measurements were conducted by one evaluator (J.Z.), blinded to the treatment groups. The areas of infiltrated white matter were then divided by the entire white matter area of the respective spinal cord section, and the result is given as relative infiltrated white matter area (in %). Representative images are shown in Figure 2C.

To analyse the spatial distribution of microgliosis in mid-sagittal brain sections from EAE^Only^ and EAE^DigiGait^ mice, sections were stained with anti-IBA1 antibodies and microgliosis sites were highlighted in a brain-template adopted from the Allen Mouse Brain Atlas [27]. Each black dot represents a single lesion per individual mouse (Figure 2D). These analyses were conducted by two evaluators blinded to the treatment groups (J.Z. and H.K.).

### 2.6. Statistical Analyses

All data are given as the arithmetic means ± SEM. Differences between groups were statistically tested using the software package GraphPad Prism 5 (GraphPad Software Inc., San Diego, CA, USA). The D’Agostino and Pearson test was applied to test for Gaussian distribution of the data. The definite statistical procedure applied for the different analyses is provided in the figure legends. *p*-value ≤ 0.05 were considered statistically significant. The following symbols are used to indicate the level of significance: * *p* ≤ 0.05, ** *p* ≤ 0.01, *** *p* ≤ 0.001, ns = not significant.

## 3. Results

### 3.1. Manipulation of DigiGait™ Does Not Decrease EAE Severity

Previous studies have shown that stress might impact on EAE disease development [28]. Since the handling of the mice during DigiGait™ recordings might lead to additional stress, in a first step we systematically compared EAE severity in MOG_35-55_-induced EAE mice which were subjected to DigiGait™ (n = 10; EAE^DigiGait^) recordings or not (n = 10; EAE^Only^). After immunization, the mice were evaluated daily for the occurrence and severity of clinical symptoms based on conventional evaluation protocols (see materials and methods section). The gait patterns were recorded daily starting six days post immunization until the mice reached an EAE score of ≥ 2 (equals hind limb paresis), or until day 16 post immunization (i.e., end of the experiment). A schematic depiction of the experimental setup is shown in Figure 2A.

As demonstrated in Figure 2B, both, EAE^Only^ and EAE^DigiGait^ mice, exhibited motor behavioral deficits which are typical for MOG_35-55_-induced EAE in C57BL/6 mice, starting with a limp tail and progressing towards hind limb paralysis. In the EAE^Only^ group, 6 out of 10 and in the EAE^DigiGait^ group 8 out of 10 mice developed clinical deficits, respectively. Although the clinical symptoms in EAE^DigiGait^ mice tended to be more severe compared to EAE^Only^ mice, no significant differences were observed for the parameters *time of disease onset* (EAE^DigiGait^, 12.38 ± 0.5650 days versus EAE^Only^, 10.83 ± 0.7923 days; *p* = 0.1286, just including mice which developed clinical disease), *maximum disease score* (EAE^DigiGait^, 1.950 ± 0.4913 days versus EAE^Only^, 1.050 ± 0.3452; *p* = 0.1512), and *cumulative disease score* (EAE^DigiGait^, 7.500 ± 2.053 days versus EAE^Only^, 4.200 ± 1.379 days; *p* = 0.1988) (Figure 2B). Next, we analyzed the extent of inflammatory infiltrates in EAE^DigiGait^ and EAE^Only^ mice to correlate functional deficits with histopathological changes. For this purpose, three spinal cord sections (cervical to lumbar level) were collected in a random fashion for each mouse and the inflamed white matter area in relation to the entire spinal cord white matter area was quantified in LFB/PAS stained sections.

As demonstrated in Figure 2C, no significant difference was observed in the extent of inflammatory demyelination between EAE^DigiGait^ and EAE^Only^ mice (EAE^DigiGait^, 23.28% ± 3.549% versus EAE^Only^, 13.99% ± 3.205%). Spearman’s correlation analysis, including data from both experimental groups, revealed a highly significant correlation between spinal cord white matter inflammation and the extent of clinical deficits (r = 0.7221; r^2^ = 0,52; 95% confidence interval = 0.59 to 0.82; *p*-value (two-tailed) ≤ 0.0001). Furthermore, we analyzed the spatial distribution of microgliosis in the brains of EAE^DigiGait^ and EAE^Only^ mice. As demonstrated in Figure 2D,E, focal microgliosis was found in diverse brain regions such as the cerebellum, dorsal midbrain (arrow in Figure 2D), ventral medulla oblongata around the inferior olivary complex (arrowhead in Figure 2D), and to some extent around the third ventricle. In summary, both cohorts demonstrate widespread CNS inflammation with no quantitative differences in the extent of CNS lesion formation.

**Figure 3:**
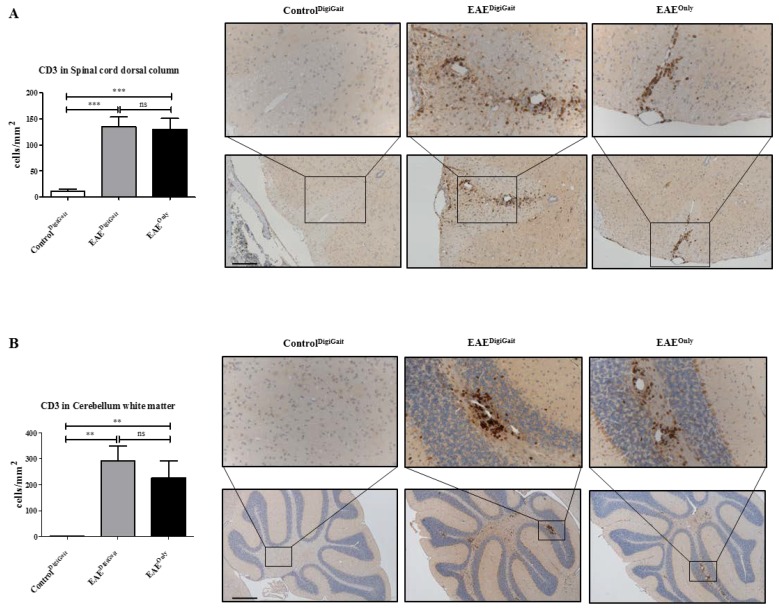
Lymphocyte densities in the spinal cord and cerebellar white matter. (**A**) Numbers of CD3^+^ lymphocytes in the dorsal column of the spinal cord (n = 75 sections) in Control^DigiGait^, EAE^DigiGait^, and EAE^Only^ mice. (**B**) Numbers of CD3^+^ lymphocytes in the white matter of the cerebellum (n = 25 sections) in Control^DigiGait^, EAE^DigiGait^, and EAE^Only^ mice. Statistical comparison was done using a one-way analysis of variance with the obtained p-values corrected for multiple testing using the Dunnett’s post hoc test. Note that no significant difference has been observed between EAE^DigiGait^ and EAE^Only^ mice. Scale bar (**A**) = 150 µm; Scale bar (**B**) = 300 µm. ** *p* ≤ 0.01, *** *p* ≤ 0.001, ns = not significant.

Beyond, we analyzed the densities of CD3^+^ lymphocytes in the spinal cord dorsal column and the white matter of the cerebellum. As demonstrated in Figure 3, lymphocyte densities were low in Control^DigiGait^, but high in EAE^DigiGait^ and EAE^Only^ mice (Spinal cord dorsal column, Control^DigiGait^ 10.87 ± 4.180 cells/mm^2^, versus EAE^DigiGait^, 134.5 ± 19.20 cells/mm^2^ versus EAE^Only^, 129.9 ± 21.00 cells/mm^2^: Cerebellum white matter, Control^DigiGait^ 1.367 ± 0.7197 cells/mm^2^, versus EAE^DigiGait^, 292.9 ± 56.61 cells/mm^2^ versus EAE^Only^, 225.9 ± 65.31 cells/mm^2^). Of note, no significant differences were observed between EAE^DigiGait^ and EAE^Only^ mice.

### 3.2. Most Gait Parameters Are Stable in Control Mice

Next, we investigated the reliability of the gait analysis procedure. To this end, gait analyses were conducted in control mice (n = 10, two separate experiments), and the coefficient of variation (CV), which is defined as the ratio of the standard deviation to the mean (SD/mean), was calculated. The term “high variability parameters” was defined as gait parameters which had a CV of higher than 30% [29]. As described in the materials and method section of this manuscript, the DigiGait™ computes 39 gait parameters for the fore limbs and 43 for the hind limbs, respectively. As listed in Table 1, 10 out of 39 (25.6%) fore limb, and 13 out of 43 (30.2%) hind limb parameters showed a high variability in control mice. This, on the one hand, indicates that not all of the gait parameters evaluated by the DigiGait^Tm^ software are adequate for the detection of a pathological gate, at least in mice at the applied experimental settings. However, a significant proportion of gait parameters (i.e., 30) can reliably be measured using the DigiGait™ apparatus.

### 3.3. Mice Show Gait Abnormalities in Hind Limbs during the EAE Pre-Clinical Phase

In a next step, we asked whether gait abnormalities can be quantified during the pre-clinical EAE phase. For this purpose, we systematically compared changes of the gait parameters in control (referred to as Control^DigiGait^; n = 10) and MOG_35-55_-immunized (referred to as EAE^DigiGait^; n = 18) mice. As it has been shown that the running speed can influence gait parameters in rodents [30], we used a constant speed of 15 cm/s.

**Figure 4:**
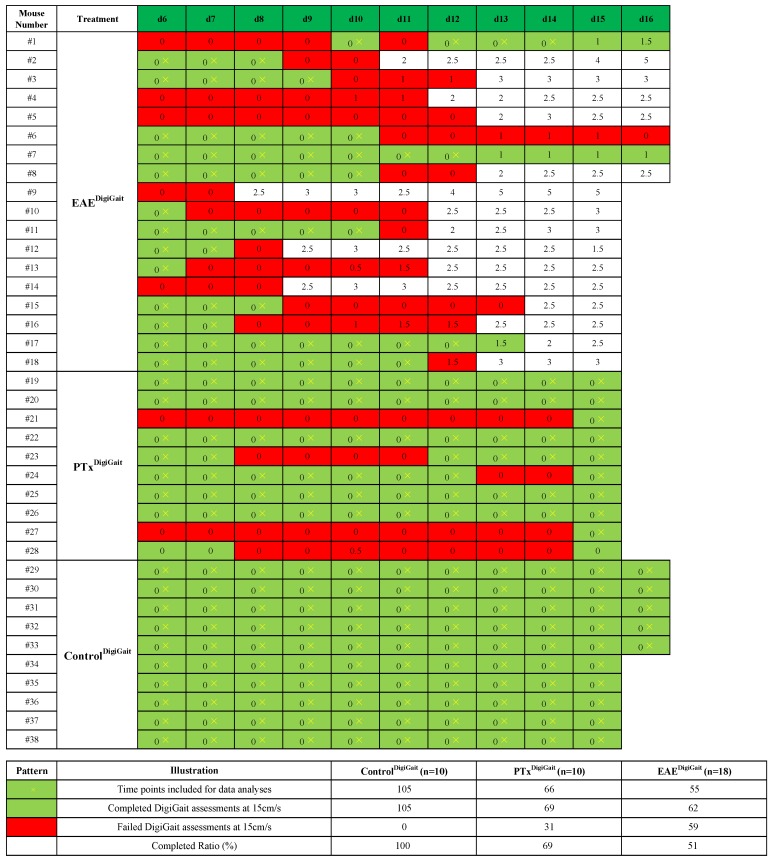
Summary of gait analyses experiments. Days with successfully conducted DigiGait^TM^ recordings are highlighted in green, whereas days on which no DigiGait^TM^ recordings could be performed are highlighted in red. Numbers in the boxes indicate the level of motor behavior deficits evaluated by classical EAE scoring. Yellow crosses indicate time points included for data analyses.

As demonstrated in Figure 4, 18 out of 20 immunized animals developed clinical EAE. EAE was severe in some animals (#2 and #9 with a score of five) but moderate in others (for example, #6 with a transient score of one). MOG_35-55_ immunization severely influenced the success rate of gait analysis recordings. Just 3 out of 18 animals could be daily evaluated until the day of disease onset (i.e., mice #7, #17, and #18), whereas 6 out of 18 animals could be daily evaluated until the day BEFORE disease onset (#3, #11, and #12, additionally to the mice #7, #17, #18). Four animals could not be evaluated at any time point after MOG_35-55_ immunization (#4, #5, #9, and #14).

As outlined in the materials and method section, gait analyses were initiated at day 6 post immunization and continued daily until the animals (i) either reached a score of ≥ 2, (ii) were not able to run on the treadmill at the given velocity (i.e., 15 cm/s), or (iii) until day 16 post immunization. Following this strategy and pooling the data from two independent experiments, 105 gait analyses were performed in control animals for the different time points with a success rate of 100%. 121 gait analyses were performed in MOG_35-55_-immunized mice with a success rate of 51% (equals 62 completed gait analyses). These results already suggest that although conventional EAE scoring protocols fail to detect overt changes (i.e., paralysis of the tail), the motor performance is already impaired at this ‘pre-clinical’ disease stage.

Blinded evaluations of the high speed ventral plane videography recordings were performed in two separate cohorts of animals, referred to as cohort#1 (five control animals and eight EAE animals) and cohort#2 (five control animals and 10 EAE animals). Only data obtained during the pre-clinical disease stages were included. In a first step, fore limb and hind limb gait parameters were evaluated in the cohort#1 mice and statistically compared. As one would expect in a model of ascending paralysis [31,32], more gait parameters were altered in the hind limbs (n = 15) compared to the fore limbs (n = 9) during the pre-clinical disease stage. As demonstrated in Table 2, 15 distinct hind limb gait metrics were found to be increased or decreased in EAE^DigiGait^ compared to Control^DigiGait^ mice during the pre-clinical disease stage. To verify these findings, the gait parameters which were found to be significantly different in the cohort#1 mice were re-evaluated in our cohort#2 mice. For the fore limb parameters, none of the 9 parameters were verified in the cohort#2 mice. In contrast, from the 15 gait parameters found to be different in the hind limbs of cohort#1 mice, 7 were verified in the second cohort. These were the gait metrics *Swing Time (Average), %Swing Stride (Average), %Stance Stride (Average), Stance/Swing (Average), Paw Angle-Left Hind, Paw Angle-Right Hind,* and *Absolute Paw Angle (Sum)*.

As demonstrated in the materials and methods section, the gait signals provided by the software requires some manual, thus subjective, adjustments. To verify that our results are indeed valid, another independent evaluator performed the analyses of cohort#1 and cohort#2 video sequences in a blinded manner. As demonstrated in Table 2, all 7 gait parameters were approved by the second evaluator.

**Figure 5:**
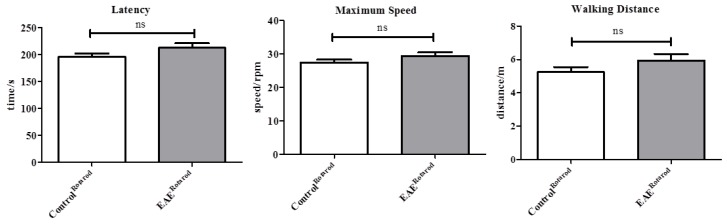
Gross locomotor ability in the Rotarod assay during pre-clinical EAE. Gait parameters were assessed in Control^Rotarod^ (n = 10) and EAE^Rotarod^ (n = 8) mice. Both cohorts were tested for their ability to run on a rotating cylinder that accelerated its speed with time (4–40 rpm in 300 s). Latencies to fall from the accelerating cylinder (i.e., *latency*), the *maximum speed* mice were able to run, and the *walking distance* on the rotating cylinder are presented as mean ± SEM. The D’Agostino and Pearson test was applied to test for normal distribution of the data. *p*-values for the effect of EAE treatment were calculated using t-test or Mann-Whitney test according to data distribution. ns = not significant.

Next, we were interested whether gait abnormalities during pre-clinical EAE can as well be detected using the Rotarod test which is widely used to evaluate the motor coordination of rodents [33,34]. To this end, performance in the rotarod test was compared between 10 control (Control^Rotarod^) mice and 8 pre-clinical EAE mice (EAE^Rotarod^). As demonstrated in Figure 5, EAE^Rotarod^ mice showed comparable values in the Rotarod parameters *latency* (EAE^Rotarod^, 212.7 ± 8.617 s versus Control^Rotarod^, 195.7 ± 7.033 s; *p* = 0.1106), *maximum speed* (EAE^Rotarod^, 29.39 ± 1.036 rpm. versus Control^Rotarod^, 27.41 ± 0.8427 rpm.; *p* = 0.1367), and *walking distance* (EAE^Rotarod^, 5.935 ± 0.3887 m versus Control^Rotarod^, 5.239 ± 0.3128 m; *p* = 0.1169) when compared with Control^Rotarod^ mice.

### 3.4. Gait Abrnoramilties in Mice Sub-Immunized with CFA and PTx

**Figure 6:**
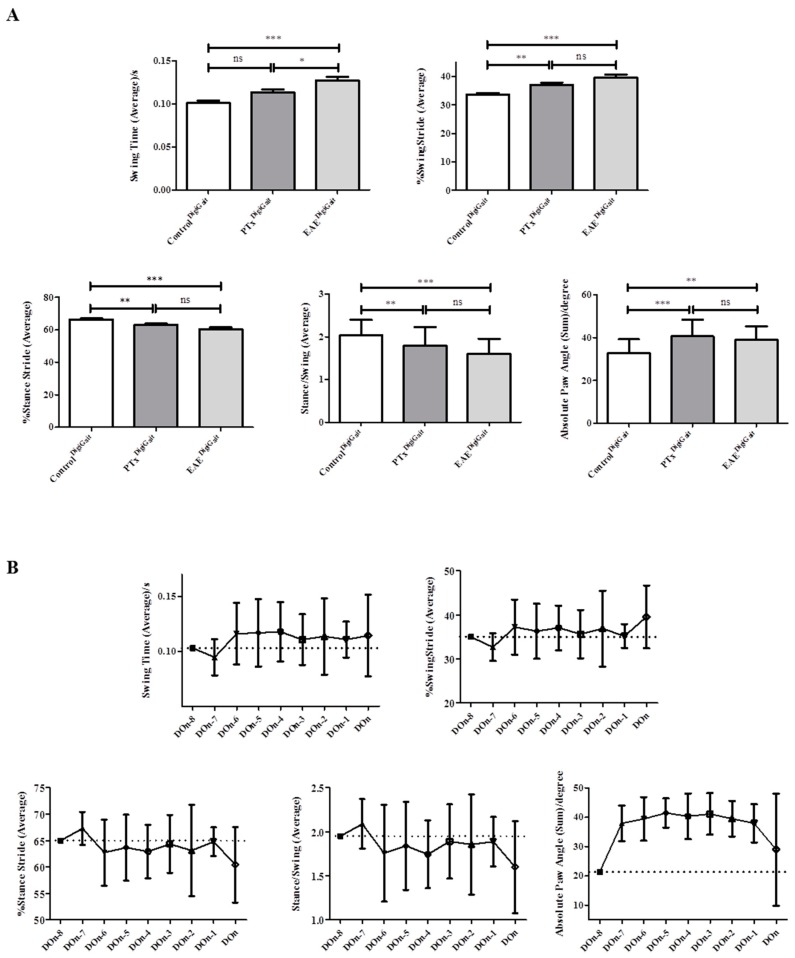
Effect of EAE sub-immunization on gait parameters. (**A**) Differences of the gait metrics in Control^DigiGait^, PTx^DigiGait^, and EAE^DigiGait^ mice. D’Agostino and Pearson test was applied to test for normal distribution of the data. *p*-values generated using one-way ANOVA with Bonferroni post-test for multiple comparisons of individual pairs of treatment. Note that the gait parameter *Swing Time* is significantly different between sub-immunized (PTx^DigiGait^) and fully immunized (EAE^DigiGait^) mice. (**B**) Gait parameters over time in fully immunized (EAE^DigiGait^) mice. Don = day of onset, Don-1 = 1 day before onset, etc. * *p* ≤ 0.05, ** *p* ≤ 0.01, *** *p* ≤ 0.001, ns = not significant.

Our analyses so far suggest that during pre-clinical EAE, motor abnormalities can be quantified using high speed ventral plane videography. Severe inflammation is characteristic for the clinical but not pre-clinical EAE phase. We, thus, assumed that diffuse, innate immune driven pathological processes account at least in part for the observed gait abnormalities. To mimic diffuse innate immune activation, we systematically investigated gait abnormalities in control mice and mice injected with CFA and PTx without the MOG_35–55_ peptide (referred to as sub-immunization). Various studies have shown that the administration of CFA and PTx without the MOG_35–55_ peptide induces diffuse innate immune activation in the CNS of mice [35,36,37,38]. In particular, we analyzed whether or not the identified gait metrics found to be altered during pre-clinical EAE are as well different in sub-immunized mice. We excluded the two parameters “paw-angle of the left hind limb” and “paw angle of the right hind limb” because both were found to be highly variable in control animals (see Table 1). Among the remaining five abnormal gait parameters during the EAE pre-clinical phase, we found that *Swing Time (Average)* was significantly different between sub-immunized (PTx^DigiGait^) and fully immunized (EAE^DigiGait^) mice. In contrast, such a difference was not observed for the other 4 gait parameters suggesting that most of the observed gait differences are due to diffuse innate immune system activation (Figure 6).

## 4. Discussion

The most commonly used behavioral evaluation method in EAE is based on the severity of motor deficits, which is mainly driven by spinal cord pathology. In most studies, each mouse is graded daily and given a score ranging from 0 to 5 [39,40,41]. Parameters include limp tail or hind limb weakness when EAE is mild and partial or complete hind limb and fore limb paralysis in severe EAE cases. Of note, this evaluation approach is neither very sensitive nor objective. Therefore, in order to detect minor motor deficits, more accurate and reliable EAE evaluation methods are urgently needed. In this study, we used the high speed ventral plane videography system DigiGait™ to characterize and quantify a set of different gait metrics during pre-clinical EAE. We were able to show (1) that manipulation during DigiGait™ measurements does not decrease EAE severity; (2) that many gait parameters are stable in control mice; (3) that the mice show hind limb gait abnormalities during pre-clinical EAE and, (4) that most of the observed gait abnormalities during pre-clinical EAE are probably driven by an interplay of innate and adaptive immune activation.

The expanded disability status scale (EDSS), which is the most commonly used measure of disability for MS, ranges from 0 to 10 in 0.5 unit increments that represent higher levels of disability and is based on the individual, subjective examination by a neurologist. EDSS steps 1.0 to 4.5 refer to people with MS who are able to walk without any aid and are based on measures of impairment in eight functional systems, among motor disturbances. Other measures of motor disability in MS include the timed 25 foot walk, which assesses ambulatory function, or walking [42], and the 9 hole peg test, which measures upper body function and manual dexterity [43]. Indeed, gait impairment is a hallmark of MS which significantly impacts on the quality of life of the individual [44]. Comparable to the human disease, MS models are characterized by gait abnormalities [4,5]. As already stated above, there is no standard EAE scoring system which research groups would use to measure EAE severity [45]. The use of different EAE scoring systems prohibits direct comparison of clinical EAE data published from different laboratories. Furthermore, the applied scoring systems rely on subjective rather than objective evaluations. An objective and quantitative approach would, therefore, be of great interest for pre-clinical trials using the EAE model.

Different automatic or semi-automatic systems have been applied to quantify gait abnormalities in different EAE models, among the CatWalk^TM^ XT system. The CatWalk^TM^ System consists of a glass walkway that is illuminated by fluorescent light. When the paw is in contact with the upper surface of the walkway, the print light is reflected, which is detected by an appropriate high speed color camera and detection software. Of note, the animal walks across the glass plate voluntarily which is different to the system applied in the current study. This method has been performed in the EAE model using different species such as Lewis rats [46], Brown Norway rats [47], or C57BL/6 mice [48]. In our study, we observed that MOG_35-55_ immunization severely influenced the success rate of gait analysis recordings. Just 3 out of 18 animals could be daily evaluated until the day of disease onset, whereas 6 out of 18 animals could be daily evaluated until the day BEFORE disease onset. This result clearly demonstrates that running at a velocity of 15 cm/s displays a motor-performance challenge which cannot be met by most of the mice during pre-clinical EAE. Bernardes et al. noted in their study that with disease progression, some animals were not able to cross the CatWalk^TM^ walkway after established EAE [48]. During pre-clinical EAE all animals were able to perform the gait analysis task which is in contrast to our results. However, one major difference between the CatWalk^TM^ and the DigiGait™ system is that in the former, mice gait is voluntary whereas in the latter, mice are forced to walk by the motorized treadmill. It is, thus, possible that forced movements are more demanding compared to voluntary ones. Nevertheless, in line with our results the authors found a decrease in *Swing Speed* which equals the observed increase of the *Swing Time* in our study.

In this study, we applied high speed ventral plane videography to analyze gait abnormalities during pre-clinical EAE. High speed ventral plane videography has been shown to be a useful approach to quantify subtle locomotors abnormalities in mouse models of neurodegenerative movement disorders, such as Amyotrophic lateral sclerosis (ALS), Huntington or cerebellar ataxia [49]. For example, altered hind limb movement, accompanied by some changes in coordination and stability characterized the gait abnormalities in SOD1 G93A transgenic mice, which is a model of ALS [49], whereas *Stride Length* and *Stride Frequencies* were found to be altered in a model of Parkinson’s disease [50]. Gait analyses were as well found to be useful in non-neurological disorders such as in collagen-induced arthritis [51] or in a model of muscular dystrophy [52]. We followed an exploratory approach (analyzing 39 different gait parameters for the fore limbs and 43 for the hind limbs) to study gait abnormalities during pre-clinical EAE. As many gait parameters change with running speed [30], the analyses were performed in this study at a constant speed of 15 cm/s (see Materials and Methods Section). Based on this extensive dataset, we identified a small set of relevant gait parameters which were different in pre-clinical EAE compared to control mice. These parameters, namely *Swing Time*, *%Swing Stride*, *%Stance Stride*, *Stance/Swing*, *Paw Angle-Left Hind*, *Paw Angle-Right Hind,* and *Absolute Paw Angle*, may be used in following studies to assess potential therapeutic effects during pre-clinical EAE. The definition of these parameters, as provided by the manufacturer of the DigiGait™ system are as follow: *Swing Time*—Time duration of the swing phase (no paw contact with belt) given in seconds; *%Swing Stride*—Percent of the total stride duration that the paw is in the air (swing phase); *%Stance Stride—*% of the total stride duration that the paw is in any contact with the belt; *Stance/Swing*—Ratio of stance phase time to swing phase time; *Paw Angle-Left or Right Hind*—The angle that the paw makes with the long axis of the direction of motion of the animal; *Absolute Paw Angle*—Absolute value of the paw angle. Having these definitions in mind it is not surprising to find the parameters *Swing Time* and *%Swing Stride* to be increased while the gait parameter *%Stance Stride* is decreased. However, this particular finding nicely demonstrates the reliability of the used evaluation method. Worth to note that, in line with our findings of an increased *Paw Angle*, these gait deficits were found to be associated with ataxia, spinal cord injury, and demyelinating disease [53].

One major finding of the present study is that gait abnormalities during the pre-clinical EAE phase can be quantified. Such alterations have as well been observed by others. For example, Leva et al. found in SJL/J mice immunized with proteolipid protein (PLP_139–151_) that the CatWalk^TM^ gait parameter *Maximum Contact Area* decreased three days post immunization, at a time point were conventional disease scoring protocols failed to detect any disease activity [54]. Similar observations were reported by Silva et al. [46], as well using the CatWalk^TM^ System in Lewis rats [46]. Of note, the gait parameter *Maximum Contact Area*, which is called *Paw Area at Peak Stance* in the DigiGait™ environment, was found to be decreased for the hind limbs in Cohort#1 animals, however, we were not able to reproduce this finding in Cohort#2 mice. In the later study, Silva et al. observed, besides a reduced *Maximum Contact Area* of the paw, reductions of the so-called *Regularity Index (RI)* during pre-clinical EAE. *RI* represents a gait metrics for motor coordination. For fully coordinated locomotion, each paw is placed exactly once every four steps. There are a total of six possible step sequence patterns that can be used by a rodent while walking. These patterns can be categorized into three groups: Alternate (Aa: [RF: Right front-RH: Right hind-LF: Left front-LH: Left hind]: RF-RH-LF-LH, Ab: LF-RH-RF-LH); cruciate (Ca: RF-LF-RH-LH, Cb: LF-RF-LH-RH); and rotary (Ra: RF-LF-LH-RH; Rb: LF-RF-RH-LH). The Ab pattern is the most commonly observed. The larger the number of missteps intersperse between regular step patterns, the lower is the *RI* [55]. The same gait parameter is not evaluated by the DigiGait™ software. However, it includes the metrics *Gait Symmetry* which computes the ratio of forelimb stepping frequency to hind limb stepping frequency. It has been shown that the parameter *Gait Symmetry* declines with age and treadmill training counteracted the decline of *Gait Symmetry* [56]. Of note, no differences with respect to the parameter *Gait Symmetry* were found during the pre-clinical EAE phase in our current study.

In a recent study, Kappos et al. analyzed the validity and reliability of the CatWalk^TM^ system as a static and dynamic gait analysis tool for the assessment of functional nerve recovery in small animal models [55]. They found that among different gait parameters, *Swing Duration* was the most reliable and valid gait parameter. In our study, *Swing Time*, which is essentially the same as *Swing Duration*, was found to be increased in both experimental cohorts and the difference was verified by two independent observers. Of note, it has been shown that *Swing Duration* increases with pain [57,58,59], and pain, which is a frequent and disabling symptom in MS patients, as well characterizes EAE animals to some extent [60]. Of note, a recent study showed that pain can as well be observed during the pre-clinical EAE phase [61]. It is, thus, possible that pain is the underlying mechanism of the observed increased *Swing Time* in our EAE mice. Further studies are needed to verify or reject this hypothesis.

Another important finding of our study is that sub-immunization of the mice with CFA and PTx is sufficient to induce moderate gait abnormalities in the experimental mice. In animal models of EAE, the disease is induced actively by immunization with myelin protein peptides, such as MOG or PLP peptide dissolved in CFA, or passively by activated neuroantigen-specific T-cells transfer. The incidence and severity of the disease induced by neuroantigens in CFA is promoted by PTx co-injection [62]. Although PTx has been widely used in EAE induction of rodents, the exact role of PTx in initiating EAE remains controversial. Historically, it was thought that this microbial product facilitates EAE by breaking down the blood-brain barrier and thereby helps pathogenic T-cells to migrate into the CNS. Further studies have shown that PTx increases the expression of endothelial adhesion molecules which triggers leukocyte infiltration into the brain [37]. PTx could also facilitate EAE induction through modulating the interaction between the innate and adaptive immune system in the response to self-antigens [36]. Moreover, PTx has other biological functions that could contribute to its activity in EAE such as inducing maturation of dendritic cells [38], enhancing T effector cells’ cytokine production as well as reducing T regulatory cells’ activity [63,64]. Murugesan et al. showed that CFA/PTx alone could cause widespread gene alterations that could prime the choroid plexus to unlock the CNS to T-cell infiltration during neuroinflammatory disease [65]. In this study, we used sub-immunization to uncover whether autoreactive T-cells are required to induce the observed gait abnormalities. As demonstrated in Figure 6A, the extent of gait alterations was found to be more severe in fully immunized mice compared to sub-immunized animals. These results suggest that both, innate and adaptive immunity, act in concert to induce gait abnormalities during pre-clinical EAE.

One major advantage of semi-automated gait analyses in EAE and other neurodegenerative diseases is that such metrics can be directly compared with measurement obtained during clinical trials. In a recent trial, Liparoti et al. investigated gait patterns in minimally disabled RRMS patients applying a three dimensional-gait analysis approach. They could show that, compared to healthy controls, RRMS show an increase of *Swing Time* [66]. Beyond, Novotna et al. were able to show that MS patients with no apparent disability (EDSS 0-1.5) showed abnormalities in the GAITRite gait analysis instrument [67], suggesting that particular aspects of human gait abnormalities can be investigated in mice.

Another important advantage of the DigiGait™ analysis system is the semi-automated analysis approach. Although some manual adjustments have to be performed during the video analysis procedure, false negative or positive results due to experimenter bias are less likely to occur. Nevertheless, blinding during the video analysis procedure is mandatory.

## 5. Conclusions

In summary, DigiGait™ is more sensitive than conventional scoring approaches to study motor deficits during the EAE pre-clinical phase. To evaluate such abnormalities we suggest to either quantify the numbers of successful runs on the treadmill and/or to quantify the gait parameters *Swing Time, % Swing Stride*, *%Stance Stride*, *Stance/Swing* ratio, or *Absolute Paw Angle*. Early detection of gait abnormalities in the EAE model may accelerate the development of therapies for MS.

## Figures and Tables

**Table 1:** High variability parameters in control mice. List of gait parameters which were found to be highly variable in control animals. High variability parameters were defined as gait metrics which show a coefficient of variation (CV) of more than 30% in control mice [29]. For more information, see the materials and methods section of this manuscript.

	Parameters with High Variability
**Fore Limbs** **(10 out of 39 parameters)** **25.6%**	Absolute Paw Angle (Sum)
	Stride Width Variability
	Step Angle Variability
	Stance Width CV
	Step Angle CV
	Paw Area Variability at Peak Stance (Average)
	Overlap Distance (Average)
	Paw Placement Positioning (Average)
	Paw Angle (Left fore limb)
	Paw Angle (Right fore limb)
**Hind Limbs** **(13 out of 43 parameters)** **30.2%**	Stride Length Variability (Average)
	Stride Width Variability
	Stride Length CV (Average)
	Stance Width CV
	Step Angle CV
	Paw Area Variability at Peak Stance (Average)
	Paw Placement Positioning (Average)
	Tau-Propulsion (Average)
	Overlap Distance (Average)
	Ataxia Coefficient (Average)
	Paw Angle (Left hind limb)
	Paw Angle (Right hind limb)
	Paw Drag (Average)

**Table 2:** Gait abnormalities during pre-clinical EAE. Summary of gait parameters found to be altered during pre-clinical EAE. Two independent experiments were performed, referred to as Cohort#1 and Cohort#2. Gait parameters were evaluated by two independent observers, referred to as Evaluator 1 and Evaluator 2. Arrows indicate whether gait metrics were increased or decreased during the pre-clinical EAE phase. During pre-clinical EAE, 9 fore limb gait parameters were found to be different in Cohort#1 mice, but not in Cohort#2 mice (indicated by ns = not significant). In contrast, 15 hind limb gait parameters were found to be different in Cohort#1 mice, and 7 of these were found to be as well different in the Cohort#2 mice (indicated by the respective *p*-value). All of these 7 parameters were verified by the Evaluator 2 (last column). The D’Agostino and Pearson test was applied to test for normal distribution of the data. *p*-values for the effect of EAE treatment were calculated using t-test or Mann-Whitney test according to data distribution. All videos were analyzed by two evaluators (J.Z. and V.Y.) blinded for the experimental groups. * *p* ≤ 0.05, ** *p* ≤ 0.01, *** *p* ≤ 0.001, ns = not significant, ↑: increased; ↓: decreased.

	Parameter Number	Parameters	Evaluator 1 Cohort#1 (Change, Significance, *p*-Value)	Evaluator 1 Cohort#2 (Change, Significance, *p*-Value)	Evaluator 2 Cohort#1 and Cohort#2 (Change, Significance, *p*-Value)
**Fore Limbs**	#1	Paw Angle Variability (Average)	↓, *, 0.0483	↓, ns, 0.7092	
	#2	Stance Width	↑, *, 0.0241	↑, ns, 0.8720	
	#3	Stride Length Variability (Average)	↓, ***, 0.0008	↑, ns, 0.5670	
	#4	Stride Width Variability	↓, ***, 0.0001	↓, ns, 0.5056	
	#5	Stride Length CV (Average)	↓, *, 0.0101	↓, ns, 0.3456	
	#6	Stance Width CV	↓, ***, < 0.0001	↓, ns, 0.6412	
	#7	Paw Area at Peak Stance (Average)	↑, *, 0.0486	↑, ns, 0.1747	
	#8	Paw Area Variability at Peak Stance (Average)	↓, **, 0.0097	↓, ns, 0.2705	
	#9	Ataxia Coefficient (Average)	↓, **, 0.0082	↓, ns, 0.2659	
**Hind Limbs**	#1	Swing Time (Average)	↑, *, 0.0239	↑, ***, < 0.0001	↑, ***,< 0.0001
	#2	%Swing Stride (Average)	↑, *, 0.0278	↑, ***, < 0.0001	↑, ***,< 0.0001
	#3	%Stance Stride (Average)	↓, *, 0.0278	↓, ***, < 0.0001	↓, ***,< 0.0001
	#4	Stance/Swing (Average)	↓, *, 0.0274	↓, ***, < 0.0001	↓, ***, 0.0002
	#5	Paw Angle-Left Hind	↑, ***, < 0.0001	↑, ***, 0.0006	↑, ***, < 0.0001
	#6	Paw Angle-Right Hind	↑, ***, < 0.0001	↑, **, 0.005	↑, ***, < 0.0001
	#7	Absolute Paw Angle (Sum)	↑, ***, < 0.0001	↑, ***, 0.0002	↑, ***, < 0.0001
	#8	Stride Width Variability	↓, **, 0.0071	↓, ns, 0.5056	
	#9	Stance Width CV	↓, **, 0.0073	↓, ns, 0.6412	
	#10	Paw Area at Peak Stance (Average)	↓, ***, 0.0005	↑, ns, 0.4922	
	#11	Paw Area Variability at Peak Stance (Average)	↓, *, 0.0403	↑, ns, 0.2317	
	#12	MAX dA/dT (Average)	↓, **, 0.0010	↑, ns, 0.2313	
	#13	Tau-Propulsion (Average)	↓, *, 0.0198	↓, ns, 0.2448	
	#14	Midline Distance (Sum)	↑, ***, < 0.0001	↓, *, 0.0442	
	#15	Paw Drag (Average)	↑, ***, 0.0001	↑, ns, 0.7821	
